# Meeting the 24-Hour Movement Guidelines and Outcomes in Adolescents with ADHD: A Cross-Sectional Observational Study

**DOI:** 10.3390/ijerph19042132

**Published:** 2022-02-14

**Authors:** Wei Wang, Justin A. Haegele, Yandan Wu, Chunxiao Li

**Affiliations:** 1 School of Physical Education & Sports Science, South China Normal University, Guangzhou 510631, China; tuboshu@m.scnu.edu.cn; 2 Department of Human Movement Sciences, Center for Movement, Health & Disability, Darden College of Education and Professional Studies, Old Dominion University, Norfolk, VA 23529, USA; jhaegele@odu.edu; 3 School of Physical Education & Sports Science, Fujian Normal University, Fuzhou 350117, China

**Keywords:** health behavior, mental health, school activity, youth, attention disorder

## Abstract

According to the 24-Hour Movement Guidelines, meeting daily recommendations for physical activity, sleep, and screen time is important for obtaining optimal health benefits. This cross-sectional observational study aimed to examine (a) the prevalence of meeting the movement guidelines; and (b) the associations between meeting the guidelines and selected outcomes in adolescents with attention-deficit/hyperactivity disorder (ADHD). Data from the 2018–2019 National Survey for Children’s Health dataset was used. Participants were adolescents (10–17 years) with ADHD and without other chronic conditions. Outcomes were flourishing, school engagement, and body weight status. Exposures of interest were adherence to the movement guidelines. The frequency of the participants’ adherence to the guidelines was estimated, and regression analyses were conducted to examine the associations between adherence to the guidelines and outcomes, adjusting for potential confounders. Complete observations were available for 634 adolescents with ADHD. Overall, 46.8% of the participants met at least one movement guideline, but only 6.5% met all three. The number of guidelines met had a significant and positive association with flourishing and school engagement (β = 0.21/0.17, *p*_trend_ < 0.001). Compared with meeting all three guidelines, significant associations with lower flourishing levels were found in participants who met none, sleep only, and sedentary time only (β = −0.38–−0.13, *p* < 0.05). Similar findings were identified in the school engagement outcome. Adherence to the guidelines was, however, not significantly associated with the odds of being overweight or obese. Collectively, the findings suggest the movement guidelines may be appropriate for extending to adolescents with ADHD and there is a need to increase adherence to the guidelines in this group.

## 1. Introduction

Attention-deficit/hyperactivity disorder (ADHD) is a neurodevelopmental disorder characterized by inattention, hyperactivity, and/or impulsivity that affects about 7.2% of children and adolescents internationally [1,2,3]. Individuals with ADHD tend to experience a range of functional deficits across settings such as employment, interpersonal relationship, and school education [3]. For example, adolescents with ADHD tend to have difficulty sustaining their attention during academic lessons, which can lead to undesirable academic performance. Persons with ADHD tend to experience increased health risk behaviors as well as decreased mental health and wellbeing [4,5]. Thus, it is crucial to identify modifiable factors that can help enhance functional and health risk behaviors among persons with ADHD.

Several volitional, health-related behaviors, such as physical activity, sedentary behavior, and sleep, have been found to be associated with physical and mental health in adolescents with and without ADHD [6,7,8,9,10,11]. In recent years, a conceptual shift has been underway where instead of considering these modifiable behaviors separately, an integrative approach suggesting “the whole day matters” has been adopted [12]. The key rationale behind this shift is that these three behaviors are codependent, and thus should be considered concurrently. As a result, the Canadian 24-Hour Movement Guidelines for Children and Youth put forward daily time recommendations for optimal health: (a) at least 60 min of moderate-to-vigorous physical activity; (b) less than 2 h of recreational screen time; and (c) 9–11 h of sleep for 5–13 years old or 8–10 h of sleep for 14–17 years old [12].

Current empirical evidence has indicated that meeting the 24-hour movement guidelines is associated with a wide range of health indicators, such as aerobic fitness, depression, and systolic blood pressure [13,14,15,16]. For example, in a cross-sectional analysis of a representative sample of 4157 Canadian children and youth, it was found that meeting more movement guidelines was associated with better overall health [13]. Similarly, in an analysis of a representative sample 20,708 adolescents from the 2016 National Survey of Children’s Health (NSCH) in the US, meeting all three 24-hour guidelines was found to associate with lower odds ratios for experiencing anxiety and depression [17]. Despite the documented benefits, only 3–10% of children and adolescents from different countries have been found to meet all three recommendations [14].

Due to the increased research attention paid to the 24-hour movement framework, a few studies have extended the framework from typical development participants to other groups, such as individuals with disabilities or chronic conditions. For example, in a cross-sectional study using data from the 2016 NSCH, less than 6% of adolescents with chronic health conditions were found to meet all three recommendations [18]. In addition, through analyzing a cross-sectional sample of 3582 adolescents who received special education services, Haegele et al. [19] found only 8.1% of them met all three guidelines, and meeting all three guidelines was associated with decreased odds of being overweight than meeting no, sleep only, or screen time only guidelines. These findings provide some initial evidence on the prevalence of meeting the 24-hour movement guidelines and associated outcomes in individuals with disabilities. To date, however, little is known about the prevalence of adherence to the 24-hour movement guidelines and outcomes in individuals with ADHD specifically.

In summary, this cross-sectional observational study aimed to: (a) examine the prevalence of adolescents with ADHD meeting the 24-hour movement guidelines; (b) the associations between meeting these guidelines and outcomes. Three outcomes were examined as part of this analysis: body weight status, school engagement, and flourishing (an indicator of overall well-being). These outcomes were selected as early research has primarily focused on examining the associations between meeting the guidelines and physical health outcomes [14]. Further, adolescents with ADHD are at increased risk of having low flourishing and school engagement levels, as well as overweight or obesity [5,20,21].

## 2. Materials and Methods

### 2.1. Study Design

A cross-sectional study design was used in the present study.

### 2.2. Data Source

We used data from the 2018–2019 NSCH combined dataset. The dataset includes a nationally representative, cross-sectional probability sample of children aged below 18 years in the US (*n* = 59,963). On behalf of the Health Resources and Services Administration, Maternal and Child Health Bureau (HRSA MCHB), the US Census Bureau conducted the two-stage survey to provide information on factors related to the well-being of children. During stage one, the US Census Bureau randomly contacted households to identify those in which children aged below 18 years lived through a screener questionnaire. If a household had more than one child, one was randomly selected. In stage two, parents or guardians who were most familiar with the identified child were invited to complete either an online or a mail-based topical questionnaire. In total, 30,530 (weighted response rate = 43.1%) and 29,433 (weighted response rate = 42.4%) surveys were collected in 2018 and 2019, respectively. More information about the combined dataset can be found in the “Fast Facts: 2018–2019 National Survey of Children’s Health” document [22]. As the used NSCH dataset is deidentified, ethical approval of the present study was exempted from the first author’s Institutional Review Board.

### 2.3. Participants

Participants included in the present study must meet the following criteria: (a) adolescents aged 10–17 years; (b) currently had ADHD as told by a doctor; and (c) had no other disability conditions or chronic diseases (e.g., autism spectrum disorder, cystic fibrosis, speech disorder). We screened and selected a subsample who met these inclusion criteria from the combined NSCH dataset. Participants who aged below 10 years, had no ADHD, reported a chronic condition in addition to ADHD, or had incomplete data were excluded for further analysis.

### 2.4. Variables and Measures

For the purpose of our study, outcome variables were flourishing, school engagement, and body weight status. Exposures of interest included the three recommended health behaviors in the 24-hour movement guidelines, which were meeting physical activity, sedentary time, and sleep duration guidelines. Potential confounders were participant demographics.

#### 2.4.1. Outcomes

Three questions (i.e., “How often does this child stay calm and in control when faced with a challenge?”; “How often does this child show interest and curiosity in learning new things?”; “How often does this child work to finish tasks he or she starts?”) were used to measure flourishing. Two questions (i.e., “How often does this child care about doing well in school?”; “How often does this child do all required homework?”) were employed to measure school engagement. Parents or guardians rated each of the items on a 4-point Likert scale (1 = “always”, 4 = “never”). Item responses were reversely coded and averaged for flourishing and school engagement, respectively. A greater mean score represented a higher level for each of the outcomes.

Proxy reports of child’s height and weight information were used to calculate body mass index (BMI). Calculation of BMI was age- and gender-specific. BMI was provided in three categories in the combined NSCH dataset: underweight—less than the 5th percentile, healthy weight—equal to 5th percentile to less than the 85th percentile, and overweight or obese—equal to 85th percentile or greater.

#### 2.4.2. Predictors

Physical activity, sedentary behavior, and sleep duration were each measured using one question. Physical activity was measured using the question, “During the past week, on how many days did this child exercise, play a sport, or participate in physical activity for at least 60 min?”. There were four options for response: “0 days”, “1–3 days”, “4–6 days”, and “every day”. Sedentary time was measured using the question, “On most weekdays, about how much time did this child spend in front of a TV, computer, cellphone, or other electronic device watching programs, playing games, accessing the internet or using social media?”. Response options included: “less than 1 h per day”, “1 h per day”, “2 h per day”, “3 h per day”, and “4 or more hours per day”. Finally, sleep was measured using the question, “During the past week, how many hours of sleep did this child get on an average weeknight?”. Options for response were: “less than 6 h”, “6 h”, “7 h”, “8 h”, “9 h”, “10 h”, and “11 or more hours”.

We created dichotomous variables (0 = “not meeting guidelines”, 1 = “meeting guidelines”) for each of these predictors based on recommendations presented by previous research. More specifically, responses of “every day”, “2 h per day or less”, and recommended age-appropriate sleep hours (i.e., 8–10 h for 14–17 years, 9 h or more for 10–13 years) were coded as meeting the physical activity, sedentary time, and sleep duration guidelines, respectively [12,23,24].

#### 2.4.3. Confounders

Potential confounders included adolescents’ age (in years), gender (male, female), severity level of ADHD (mild, moderate/severe), medication status (yes, no), race (White, Black/African, other), household income level (≤0–99% federal poverty level, ≥100% federal poverty level), and highest household education level (<college degree, ≥college degree). These variables were included as they were potentially related to health behaviors, flourishing, school engagement, and BMI [19,21,25,26,27].

### 2.5. Statistical Analyses

We used descriptive statistics (mean and SD, or frequency and percentage) to present participant demographics, adherence to each of the 24-hour movement guidelines and specific combinations, flourishing, school engagement, and body weight status. Two multiple regression analyses were conducted to determine the associations between flourishing/school engagement and (a) number of guidelines met; and (b) specific combinations of guidelines. A binary logistic regression model was generated to estimate the odds of being overweight or obese as a function of (a) number of guidelines met; and (b) specific combinations of guidelines. We focused on the comparison between healthy weight and overweight/obese. We conducted trend analyses to examine whether meeting more guidelines was associated with better outcomes. Regression analyses were adjusted for potential confounders, including adolescents’ age, gender, severity level of ADHD, medication status, race, household income level, and highest household education level. We conducted all analyses with SPSS (Version 25, IBM; Armonk, NY, USA). A statistical significance level of 0.05 was set.

## 3. Results

### 3.1. Sample Characteristics

First, we selected those participants (*n* = 658) who met the listed eligibility criteria. We then excluded some participants (*n* = 24) who had incomplete observations. The sample that remained for analyses consisted of 634 adolescents with ADHD. The mean age of the sample was 13.76 years (SD = 2.20) and 70.4% were male. Over half of the sample had mild ADHD (66.2%) and were on medication for treating ADHD symptoms (63.6%), and the majority of them were White (87.2%). Only a small portion of the sample was at 0–99% of federal poverty level (7.3%) and 60.4% held a college degree or above. Around one-fourth of the sample was overweight or obese (see Table 1).

### 3.2. Adherence to the Movement Guidelines

Overall, 18.1%, 46.8%, 28.5%, and 6.5% of the sample met no guidelines, one out of three, two out of three, and all three 24-hour movement guidelines, respectively. Of those who met only one guideline, meeting “sleep only” was the most prevalent (29.5%). For those who met two recommendations, meeting “sleep + screen time only” was the most prevalent (20.8%) (see Table 1).

### 3.3. Meeting Guidelines and Outcomes

Table 2 presents the associations between meeting movement guidelines and outcomes. The results of multiple regression analysis indicated that there was a positive and significant association between the number of guidelines met and flourishing (β = 0.21, *p*_trend_ < 0.001). Compared with meeting all three guidelines, meeting none or only one guideline was significantly associated with a lower flourishing level (β = −0.28/−0.32, *p* < 0.001). Compared with meeting all three guidelines, meeting “sleep only” and “sedentary time only” had negative and significant associations with flourishing (β = −0.38/−0.13, *p* < 0.05). These models accounted for 13–16% of the total variance in flourishing.

Regarding school engagement, this outcome had a positive and significant association with the number of guidelines met (β = 0.17, *p*_trend_ < 0.001). Compared with meeting all three guidelines, meeting none or only one guideline was significantly associated with a lower level of school engagement (β = −0.25/−0.21, *p* < 0.01). Further, compared with meeting all three guidelines, meeting “sleep only” or “physical activity + sleep only” was negatively and significantly associated with school engagement (β = −0.25/−0.12, *p* < 0.05). These models accounted for 13–14% of the total variance in school engagement.

As for BMI, the results of binomial logistic regression showed no significant associations between the number of guidelines met and odds of being overweight/obese. Their dose-response relationship, however, was significant based on the trend analysis (OR = 0.79, *p*_trend_ = 0.04). Lastly, none of the associations between the specific combinations of guidelines met and odds of being overweight/obese was significant.

## 4. Discussion

As an extension of early research, our cross-sectional analysis examined the 24-hour movement framework in a nationally representative sample of US adolescents with ADHD. We found that only a small proportion of the sample (6.5%) met all three guidelines. We also found, for the first time, a significant and favorable association between number of guidelines met and two of the examined outcomes (i.e., flourishing and school engagement). However, adherence to the guidelines and odds of being overweight/obese was not significantly associated with each other. Our findings contribute to the broader literature on the 24-hour movement framework and address the attempt by DiPietro et al. to understand movement behaviors among disability groups [28].

### 4.1. Compliance with 24-Hour Movement Guidelines

Of concern, 18.1% of the adolescents with ADHD in this analysis met none of the 24-hour movement guidelines. Further, only 6.5% of the sample met all three guidelines. These low figures are consistent with other prior works. For example, in a recent study of 54 Canadian youth with sensory and physical disabilities, Arbour-Nicitopoulos et al. [29] found that 18.5% of the participants did not meet any of the guidelines and 3.7% met all three guidelines. Similarly, Healy et al. [18] conducted a cross-sectional analysis of 2016 NSCH data, and found that 18.7% of the youth with chronic health conditions (*n* = 24,405) met none of the guidelines and only 5.4% of the sample met all three guidelines. Taken together, our study and others suggested that adolescents with ADHD and other chronic health conditions fall far short of the suggested movement guidelines [12]. Thus, interventions to enhance compliance with the guidelines are needed for adolescents with disabilities. For our sample, meeting daily physical activity appears to be of particular concern and should be enhanced, given that only 17.8% of the sample met the physical activity guideline of participating in physical activities for 60 min per day.

### 4.2. Movement Guidelines and Flourishing

A key strength of our study was the consideration of outcomes other than physical health. Our study provided initial evidence that the number of movement guidelines met was significantly and positively associated with flourishing in adolescents with ADHD. This finding is in line with a recent meta-analytic research, in which a dose-response association between the number of guidelines met and another psychological health indicator, depression, was evident in children and adolescents without disabilities [14]. Our study also found that meeting all three movement guidelines was associated with greater flourishing compared with meeting none of the guidelines. A similar finding was observed in a cross-sectional observational study, in which adults with visual impairments achieving all three movement guidelines reported a higher level of health-related quality of life than those met none of the guidelines [30]. These findings are somewhat expected given recent systematic reviews have shown that each of the individual movement behaviors are associated with mental health indicators among adolescents without disabilities [7,8,11]. Interestingly, we found meeting “sleep only” or “sedentary time only” (but not “physical activity only”) was associated with a lower level of flourishing. The finding implies that these three movement behaviors are not equally important for facilitating flourishing, and it seems that meeting the physical activity guideline plays a more significant role than achieving each of the other two guidelines. Indeed, physical activity has been a commonly explicated determinant of human flourishing in the extant literature [31].

### 4.3. Movement Guidelines and School Engagement

School engagement was another outcome of exposure to movement behaviors that was a unique consideration in our study. In prior research, sleep and physical activity have been shown to individually predict school engagement in adolescents with ADHD [10,32]. As an extension of previous findings, a dose-response association between the number of movement guidelines met and school engagement was observed in this analysis. Similarly, in a cross-sectional analysis with 4524 US children without disabilities, Walsh et al. [15] found a positive association between number of movement guidelines met and global cognition, a protective factor of school engagement. In theory, limiting sedentary time, being active, and securing adequate sleep duration appear to enhance executive function, which might help to improve school engagement in individuals with ADHD [33,34].

We also observed that meeting all three guidelines was associated with greater school engagement than meeting none, “sleep only”, or “physical activity + sleep only”. This finding suggests that the three movement behaviors do not act in isolation, and it is highly possible that meeting sedentary time alone or in combination with other individual movement behaviors is of significance for school engagement. In relation to our study findings, few studies have examined which combinations of guidelines are most beneficially related to academic outcomes. For example, Lien et al. [35] reported that adolescents without disabilities who met all three guidelines, “screen time only”, or “sleep guideline only” had better overall academic performance than those who did not meet any of the guidelines. Similarly, Watson et al. [36] found that adolescents without disabilities meeting either all three guidelines or “screen time + sleep only” reported better literacy achievement. Our findings extend the previous research and speak to the relevance of using the 24-hour movement framework in understanding school-related outcomes in adolescents with ADHD.

### 4.4. Body Weight Status

The final outcome variable considered in the present study was body weight status. Approximately one in four adolescents with ADHD was either overweight or obese in this analysis. Interestingly, although the rate was high, the figure was lower than that in adolescents without disabilities (30.2%) or adolescents receiving special education services (37.8%) in prior analyses of the NSCH [16,19]. These differences may be due to the different datasets being used for comparisons between ours and these two studies (2018–2019 NSCH vs. 2016–2017 NSCH dataset). Symptoms of hyperactivity may also explain the difference as this ADHD characteristic may increase energy expenditure. Paradoxically, the inattention and impulsivity that characterize ADHD may cause dysregulated eating behaviors and subsequently lead to overweight or obese [37]. More studies are therefore needed to explore possible explanations.

Although the trend analysis was significant, we found neither the number of guidelines met nor specific combinations of guidelines met was significantly associated with odds of being overweight or obese in adolescents with ADHD. These findings are similar to a recent study, which showed that meeting all three guidelines did not contribute to lower odds of being overweight or obese than meeting other specific combinations of guidelines in adolescents receiving special education services [19]. However, in studies with adolescents without disabilities, meeting all three guidelines was associated with lower odds of overweight or obesity than meeting none or one guideline [13,16,38]. The discrepancy suggests associations between movement behaviors and overweight/obesity are less clear in adolescents with disabilities. Other factors, such as diet and medication use, may play a more significant role in contributing to weight gain in adolescents with certain disabilities than the three movement behaviors [19]. Medication use however was not found to associate with odds of being overweight or obese in our study. Thus, further investigations are needed to understand predictors of body weight status in adolescents with ADHD.

### 4.5. Limitations and Future Directions

Despite the novel findings and contributions discussed above, our study has several limitations. First, although the NSCH dataset consists of a nationally representative probability sample, it is based on a proxy report that is prone to response bias. For example, proxies have a tendency to report more health or functional limitations than disability groups, especially for private, unobservable, and complex questions [39]. Thus, objective or self-reported approaches might be used for measuring outcomes that are unobservable or complex in future. Second, our study group is limited to adolescents with ADHD and without other chronic conditions. Thus, the findings may not be generalized to other groups, such as children with ADHD and adolescents with more than two disability conditions. On the other hand, focusing on our study group is beneficial as other chronic conditions may confound these findings. Future work is recommended to examine whether the present findings can be replicated in other study groups. Third, the questions asked in the NSCH failed to provide adequate information for some of our study variables. For example, the question did not capture specific intensities of physical activity. As a result, we are not able to qualify the contributions of light, moderate, or vigorous intensity physical activity to targeted outcome variables. Additional questions or objective measures (e.g., accelerometers) can be used to obtain more specific information for the study variables in future. Finally, the cross-sectional analysis limits our ability to delineate bi-directional associations between compliance with 24-hour movement guidelines and outcomes. Future prospective research is needed to examine the associations over a period.

## 5. Conclusions

To conclude, although we did not find a significant association between adherence to the 24-hour movement guidelines and odds of being overweight or obese, meeting more movement guidelines was significantly associated with increased flourishing and school engagement among US adolescents with ADHD. In general, the greatest benefit was seen with meeting all three recommendations compared with meeting none or only one guideline. Only a small proportion of the study group, however, was in compliance with the movement guidelines. Collectively, our findings suggest the guidelines may be appropriate for extending to adolescents with ADHD. Stakeholders, such as parents and educators, should consider promoting movement behaviors in this group.

## Figures and Tables

**Table 1:** Participant characteristics.

Characteristics	Value ^2^
Age (years) (10–17) ^1^	13.76 (2.20)
Gender	
Male	448 (70.7%)
Female	186 (29.3%)
Severity level of ADHD	
Mild	420 (66.2%)
Moderate or severe	214 (33.8%)
Medication status	
Yes	403 (63.6%)
No	231 (36.4%)
Race	
White alone	553 (87.2%)
Black or African American alone	27 (4.3%)
Other	54 (8.5%)
Household poverty level	
≤0–99% federal poverty level	46 (7.3%)
≥100% federal poverty level	588 (92.7%)
Highest household education level	
<College degree	251 (39.6%)
≥College degree	383 (60.4%)
Physical activity participation (min/day)	
≥60	113 (17.8%)
<60	521 (82.2%)
Average weeknight sleep	
Meet age-appropriate hours	391 (61.7%)
Below age-appropriate hours	243 (38.3%)
Weekday screen time viewing (h/day)	
≤2	278 (43.8%)
>2	356 (56.2%)
Number of movement guidelines meet	
None	115 (18.1%)
One out of three	297 (46.8%)
Physical activity only	23 (3.6%)
Sleep only	187 (29.5%)
Screen time only	87 (13.7%)
Two out of three	181 (28.5%)
Physical activity + sleep only	31 (4.9%)
Physical activity + screen time only	18 (2.8%)
Sleep + screen time only	132 (20.8%)
All three	41 (6.5%)
Flourishing (1–4) ^1^	3.03 (0.53)
School engagement (1–4) ^1^	3.13 (0.68)
Body weight status	
Underweight	54 (8.5%)
Normal weight	428 (67.5%)
Overweight or obese	152 (24.0%)

Note: ADHD = attention deficit hyperactivity disorder. ^1^ Possible range. ^2^ Values are mean ± SD or *n* (%).

**Table 2:** Associations between meeting movement guidelines and outcomes.

	Flourishing		School Engagement		Body Weight Status ^1^	
	β (95%CI) ^2^	*p*	β (95%CI) ^2^	*p*	OR (95%CI) ^2^	*p*
Number of guidelines meet						
None	−0.28 (−0.41, −0.15)	<0.001	−0.25 (−0.38, −0.12)	<0.001	2.06 (0.85, 5.04)	0.11
One of three	−0.32 (−0.47, −0.16)	<0.001	−0.21 (−0.36, −0.05)	0.009	1.43 (0.62, 3.31)	0.40
Two of three	−0.12 (−0.27, 0.02)	0.10	−0.11 (−0.26, 0.03)	0.13	1.22 (0.51, 2.91)	0.66
All three	Reference		Reference		Reference	
Trend analysis	0.21 (0.13, 0.28)	<0.001	0.17 (0.09, 0.24)	<0.001	0.79 (0.62, 0.99)	0.04
*R* ^2^	0.13		0.13		NA	
Specific combinations of guidelines meet						
None	−0.28 (−0.41, −0.15)	<0.001	−0.25 (−0.38, −0.12)	<0.001	2.10 (0.86, 5.12)	0.11
Physical activity only	−0.04 (−0.13, 0.05)	0.39	−0.01 (−0.10, 0.08)	0.76	0.32 (0.06, 1.70)	0.18
Sleep only	−0.38 (−0.52, −0.23)	<0.001	−0.25 (−0.39, −0.10)	0.001	1.42 (0.60, 3.39)	0.43
Sedentary time only	−0.13 (−0.24, −0.01)	0.04	−0.09 (−0.21, 0.03)	0.13	2.00 (0.79, 5.03)	0.14
Physical activity + sleep only	−0.09 (−0.18, 0.004)	0.06	−0.12 (−0.21, −0.02)	0.02	1.56 (0.49, 4.97)	0.45
Physical activity + sedentary time only	−0.03 (−0.11, 0.06)	0.52	−0.02 (−0.11, 0.07)	0.66	0.64 (0.12, 3.43)	0.60
Sleep + sedentary time only	−0.11 (−0.24, 0.03)	0.12	−0.08 (−0.22, 0.05)	0.23	1.24 (0.50, 3.06)	0.64
All three	Reference		Reference		Reference	
*R* ^2^	0.16		0.14		NA	

Note: CI = confidence interval. β = standardized beta coefficients. OR = odds ratio. NA = not available. ^1^ Healthy weight is used as a reference category (healthy weight vs. overweight/obese). ^2^ All models are adjusted for age, gender, severity level of ADHD, medication status, race, household poverty level, and highest household education.

## Data Availability

Data supporting reported results is available from the corresponding authors upon request.
